# Evaluation of twin-arginine translocation substrate proteins as potential antigen candidates for serodiagnosis of brucellosis

**DOI:** 10.3389/fvets.2025.1398983

**Published:** 2025-02-05

**Authors:** Yao Wu, Xin Yan, Mingjun Sun, Xiaohan Guo, Jiaqi Li, Xiangxiang Sun, Mengda Liu, Haobo Zhang, Wenlong Nan, Weixing Shao, Fangkun Wang, Xiaoxu Fan, Shufang Sun

**Affiliations:** ^1^Laboratory of Zoonoses, China Animal Health and Epidemiology Center, Qingdao, China; ^2^College of Animal Science and Veterinary Medicine, Shandong Agricultural University, Tai'an, China; ^3^Key Lab of Environment and Health, School of Public Health, Xuzhou Medical University, Xuzhou, China; ^4^Key Laboratory of Animal Biosafety Risk Prevention and Control (South), Ministry of Agriculture and Rural Affairs, Qingdao, China; ^5^Key Laboratory of Ruminant Infectious Disease Prevention and Control (East), Ministry of Agriculture and Rural Affairs, Qingdao, China

**Keywords:** *Brucella*, twin-arginine protein translocation, translocated substrates, diagnosis, ELISA

## Abstract

**Introduction:**

Brucellosis, an infectious zoonotic disease caused by members of the genus *Brucella*, results in chronic multi-organ injury. Improving the specificity and sensitivity of serological methods for diagnosing brucellosis necessitates the development of novel diagnostic antigens. The twin-arginine translocation (Tat) pathway is responsible for transporting folded proteins across the cytoplasmic membrane and has been implicated in the virulence of *Brucella*. Three Tat substrate proteins—L,D-transpeptidase ErfK (A0577), linear amide C-N hydrolase YxeI (A1479), and thioesterase domain-containing protein EntF (B0249)—contribute significantly to *Brucella* virulence. However, the roles of these Tat substrate proteins in diagnosing brucellosis remain unclear.

**Methods:**

In this study, ErfK, YxeI, and EntF were expressed in prokaryotic cells and utilized as diagnostic antigens. The clinical sera from bovines and sheep diagnosed with brucellosis were analyzed using indirect ELISA with these proteins.

**Results:**

For bovine serum, the combined protein group (ErfK + YxeI + EntF) and YxeI demonstrated the highest diagnostic accuracy of 94.23% and 93.58%, respectively. Meanwhile, the combined protein group showed the strongest ability to detect *Brucella* in sheep serum, achieving an accuracy of 88.10%. Both the combined protein group and YxeI displayed no cross-reactivity with rabbit serum immunized against *Yersinia enterocolitica* O9, *Escherichia coli* O157:H7, *Mycobacterium tuberculosis*, *Vibrio cholerae*, *Legionella*, and *Salmonella*, indicating relatively good specificity.

**Conclusion:**

The findings of this study suggest that Tat substrate proteins serve as promising candidate antigens with significant potential value for the clinical diagnosis of brucellosis.

## 1 Introduction

Brucellosis is a significant global zoonotic disease caused by bacteria of the genus *Brucella*. This disease is characterized by bacterial persistence within the reticuloendothelial system, and infection can lead to abortion in infected ruminants (1, 2). Zoonotic transmission of *Brucella* to humans occurs through direct contact with infected animals or consumption of *Brucella*-contaminated foods, such as meat, milk, and dairy products (3, 4). In humans, brucellosis is manifested by weakness, undulant fever, and chronic inflammation in various organs, posing a serious threat to human health and wellbeing (5). A crucial step in controlling this disease is the accurate diagnosis and elimination of infected animals, underscoring the need for enhanced timely and precise diagnostic measures for brucellosis.

Several diagnostic tools are employed for the detection of brucellosis. The gold standard for diagnosis is the isolation of the bacterium from blood, tissue specimens, body fluids, and bone marrow; however, the isolation frequency is often low, and the results are not obtained immediately. Consequently, serological tests are commonly used for diagnosing brucellosis in both humans and animals. Classical serological tests—such as the Rose Bengal plate agglutination test (RBPT), tube agglutination test (SAT), fluorescence polarization assay (FPA), and complement fixation test (CFT)—detect antibodies against lipopolysaccharide (LPS). These methods, however, may cross-react with other Gram-negative bacteria, such as *Yersinia enterocolitica* O9 and *Escherichia coli* O157:H7, leading to incorrect diagnoses (6, 7). The limitations of classical serological tests highlight the urgent need to identify alternative antigens for brucellosis detection.

Among the non-polysaccharide *Brucella* cell protein antigens, the type IV secretion system component protein VirB12 and the outer membrane proteins Omp31, Omp25, and the 26 kDa periplasmic immunogenic protein bp26 show promise as useful serological diagnostic markers for brucellosis (8–10). In our previous study, we found that the twin-arginine translocation (Tat) system and its substrates for *B. melitensis* M28 are crucial for *ex vivo* and *in vivo* infections; however, the role of Tat substrate proteins in diagnosing brucellosis remains unclear. This study investigates three *Brucella* virulence-related Tat substrate proteins—L,D-transpeptidase ErfK (A0577), linear amide C-N hydrolase YxeI (A1479), and thioesterase domain-containing protein EntF (B0249)—to systematically analyze their roles in the diagnosis of brucellosis in bovine and sheep sera.

## 2 Methods

### 2.1 Serum samples

A total of 156 bovine sera (brucellosis-positive sera = 43, brucellosis-negative sera = 113) and 126 sheep sera (brucellosis-positive sera = 34, brucellosis-negative sera = 92) were provided by the China Animal Health and Epidemiology Center (Qingdao, China). All brucellosis-positive sera were confirmed by the Rose Bengal plate agglutination test (RBPT) and tube agglutination test (SAT). These seropositive sera were obtained from animals confirmed by bacteriological identification for *Brucella*. Negative sera were originated from a brucellosis-free area in China. The bovine and sheep negative and positive control sera for brucellosis were purchased from the China Veterinary Drug Control Institute for antigenicity analysis and indirect enzyme-linked immunosorbent assay (iELISA).

### 2.2 Cloning, expression of the target genes, and protein purification

The reference DNA sequence of *Brucella melitensis* M28 (NCBI reference numbers are NC_017244.1 and NC_017245.1 for chromosome 1 and chromosome 2, respectively) was used for designing primers, comparing amplified sequences, and so on. In our study, three Tat substrate proteins, namely, ErfK (A0577, Accession: WP_002963714.1), YxeI (A1479, Accession: WP_002964574.1), and EntF (B0249, Accession: WP_002968302.1), were selected, which are related to *Brucella* virulence. The DNA fragments of ErfK, YxeI, and EntF, synthesized by Sangon Technology Co., Ltd. (Shanghai, China), were inserted into the expression vector pET30a. Expression and purification of the recombinant proteins were performed in our laboratory as described elsewhere (4).

### 2.3 Western blot

Western blot analysis of antigens was performed as previously described (7). Following separation by SDS-PAGE, the target proteins were transferred to a nitrocellulose membrane. The membrane was blocked with 5% skim milk in phosphate-buffered solution (PBS) for 1 h at room temperature, washed three times with PBST (PBS containing 0.5% Tween-20), and incubated with bovine and sheep brucellosis-positive sera (1,1,000) overnight at 4°C. The membrane was washed with PBST three times and incubated with horseradish peroxidase (HRP)-labeled protein G (Thermo, USA) used at a 1:8,000 dilution. Finally, the membrane was washed with PBST three times, and development of the HRP signal was performed using an electrochemiluminescence (ECL) reagent (Yeasen, China).

### 2.4 Different antigens and combinations

The expressed proteins rErfK, rYxeI, and rEntF were analyzed for their efficiency in detecting bovine and sheep brucellosis. The different antigens and combinations are shown in Table 1.

**Table 1:** Different tat substrate protein antigens.

Antigens	Proteins
Single antigen	rErfK (A0577)
Single antigen	rYxeI (A1479)
Single antigen	rEntF (B0249)
Combination	rErfK + rYxeI + rEntF (1:1:1)

### 2.5 Establishment of the indirect ELISA method

*Brucella* antibodies in bovine and sheep sera were detected by indirect ELISA. For the procedure, the Tat substrate-coated antigen was produced by a combination of equal concentrations of rErfK, rYxeI, and rEntF and used as one coating antigen. 100 μL of the combined protein group and purified single recombinant Tat substrate proteins at a final concentration of 0.78 μg/mL in 0.05 M bicarbonate buffer were added to each well in a 96-well plate (NUNC, Denmark), and the plate was incubated for 1 h at 37°C. After washing three times with PBST, the plate was blocked in 5% skim milk in PBS for 90 min at 37°C. Next, the plate was washed three times with PBST, 100 μL of serum was added to each well, and the plate was incubated for 2 h at 37°C. Subsequently, the plate was washed three times with PBST, HRP-labeled protein G was added (1,8,000 dilution, PBS) (Thermo, USA), and the plate was incubated at 37°C for 1 h. After washing three times with PBST, 100 μL tetramethylbenzidine (TMB) substrate solution (Sigma, USA) was added to each well for 5 min at room temperature in complete darkness. Then, the reaction was stopped by adding 50 μL of ELISA Stop Solution (Solarbio, China). Finally, the OD_450_ was measured using an ELISA plate reader (TECAN, Switzerland).

Dot plots, receiver operating characteristic (ROC) curves, and the area under the ROC curve (AUC) were obtained to determine the effectiveness of single Tat substrate antigens and combined antigens (22). A cutoff value was calculated by the Youden index (specificity + sensitivity - 1), and true positives (TP), true negatives (TN), false positives (FP), false negatives (FN), accuracy, positive predictive value (PPV), and negative predictive value (NPV) were also obtained. Accuracy, PPV, and NPV were calculated according to the following equations: Accuracy = (TP + TN/TP + FN + TN + FP) × 100; PPV = (TP/TP + FP) × 100; and NPV = (TN/TN + FN) × 100 (11).

### 2.6 Specificity and repeatability assessment

To verify the specificity of the three proteins as diagnostic antigens, we analyzed rabbit serum that had been immunized with pathogenic bacteria that were prone to cross-reaction with *Brucella*, including *Yersinia enterocolitica* O9, *Escherichia coli* O157:H7, *Mycobacterium tuberculosis*, *Vibrio cholerae*, *Legionella*, and *Salmonella*, which was purchased from Tianjin Biochip Corporation (Tianjin, China). The serum was selected for the cross-reaction assay using HRP-labeled sheep anti-rabbit IgG secondary antibody (1:5,000) (Thermo, USA). To estimate the inter- and intra-assay precision, the coefficients of variation (CVs) of repeated tests within batches and between batches, respectively, were all calculated five times by using the positive and negative brucellosis serum samples (12). The detection procedures were the same as those described in section serum testing.

### 2.7 Statistical analysis

Dot plot and receiver operating characteristic (ROC) analyses were performed using GraphPad Prism version 10.0.0. A *p*-value of <0.05 was considered to be statistically significant.

## 3 Results

### 3.1 Expression and purification of tat substrate proteins

**Figure 1:**
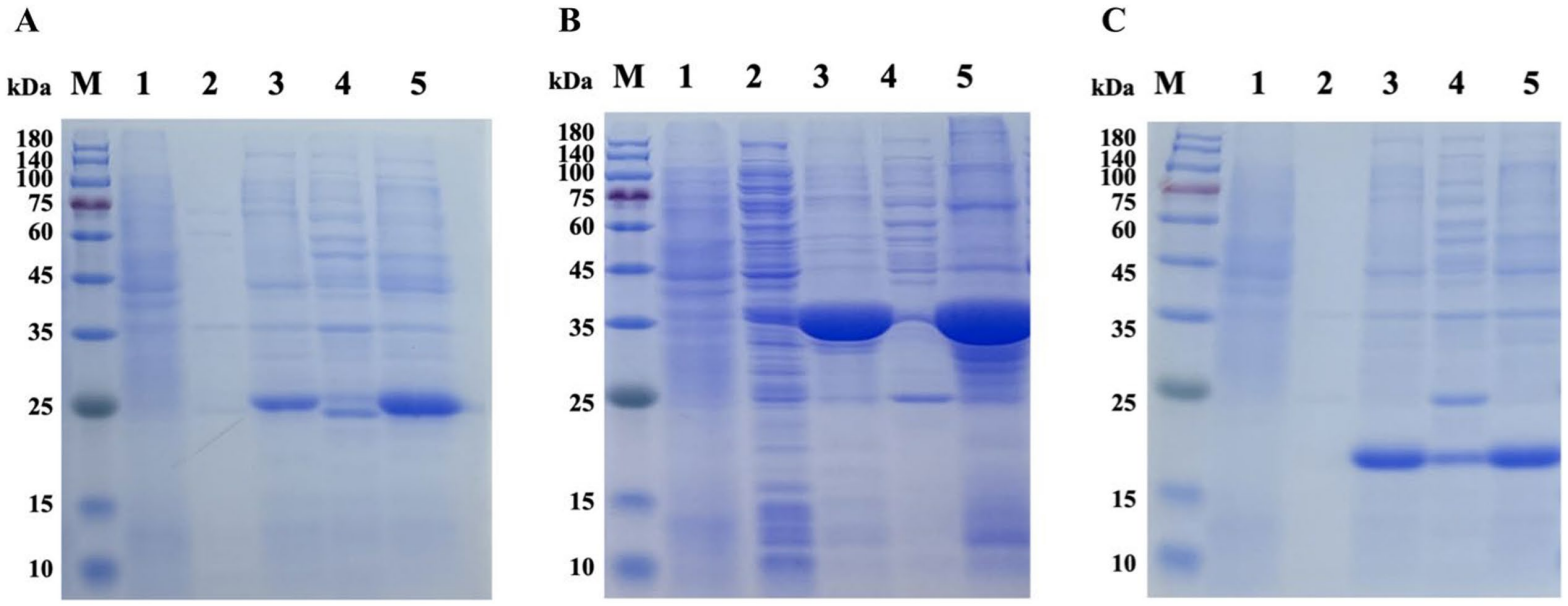
Prokaryotic expression of recombinant proteins. Lane M: protein marker; Lane 1: non-induced ErfK **(A)**, YexI **(B)**, and EntF **(C)**; Lane 2: induced ErfK **(A)**, YexI **(B)**, and EntF **(C)** supernatant at 20°C; Lane 3: induced ErfK **(A)**, YexI **(B)**, and EntF **(C)** precipitate at 20°C; Lane 4: induced ErfK **(A)**, YexI **(B)**, and EntF **(C)** supernatant at 37°C; and Lane 5: induced ErfK **(A)**, YexI **(B)**, and EntF **(C)** precipitate at 37°C.

**Figure 2:**
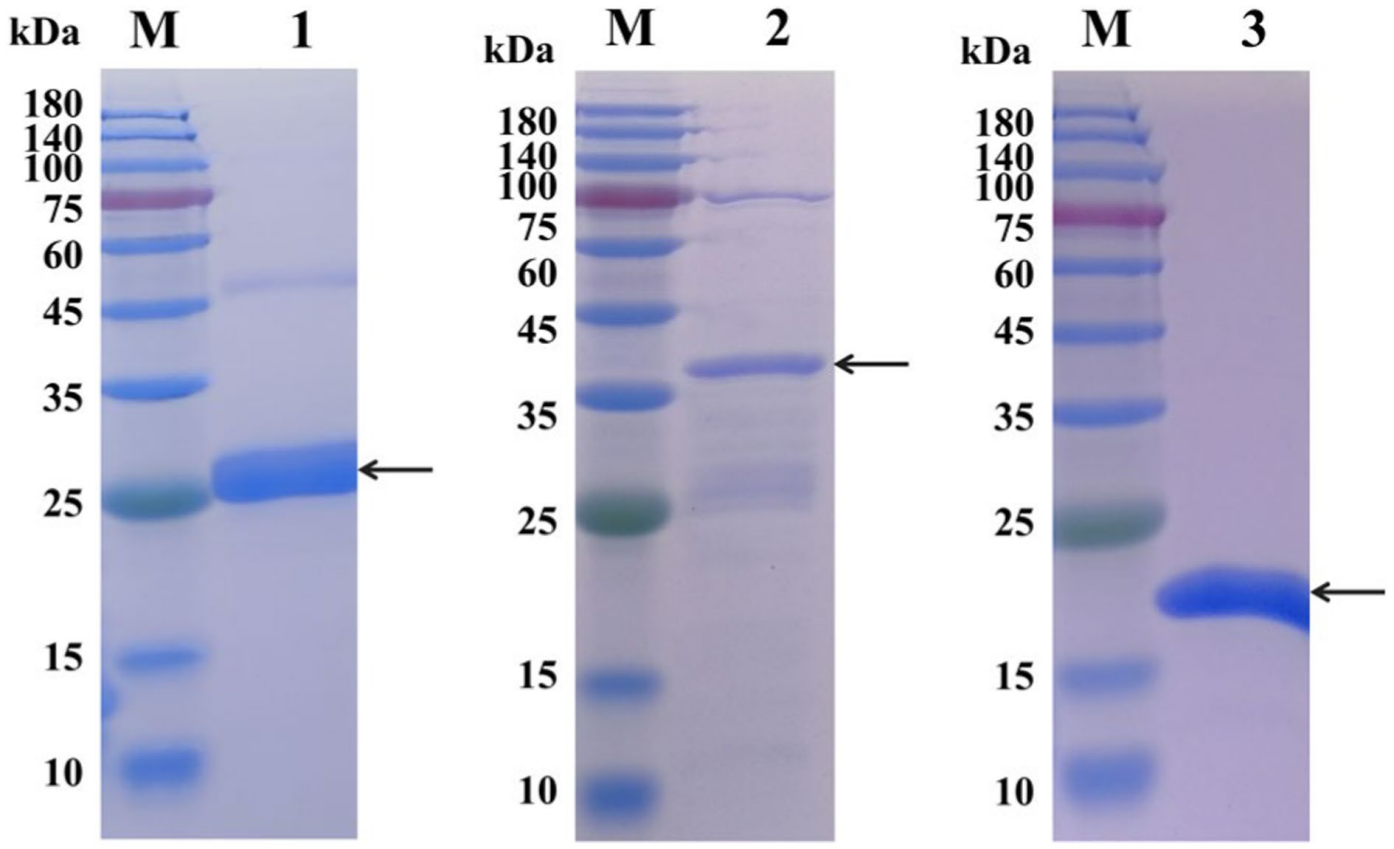
SDS-PAGE analysis of the purification of recombinant proteins. Lane M: protein marker; Lane 1: rErfK protein (25 kDa); Lane 2: rYxeI protein (38 kDa); and Lane 3: rEntF protein (21 kDa).

Tat substrate proteins ErfK, YxeI, and EntF were expressed and purified using a previously described hybrid procedure of denaturing–renaturing procedure (13, 14). The SDS-PAGE analysis showed that the majority of the expressed three Tat substrate proteins were found in the inclusion body (Figure 1), and the Ni-NTA Spin Kit purified the inclusion body. After expression and purification, SDS-PAGE confirmed that the three Tat substrate proteins were successfully purified, with the molecular weights of rErfK, rYxeI, and rEntF being 25 kDa, 38 kDa, and 21 kDa, respectively (Figure 2).

### 3.2 Antigenicity assessment of tat substrate proteins by reacting with sera using Western blot

**Figure 3:**
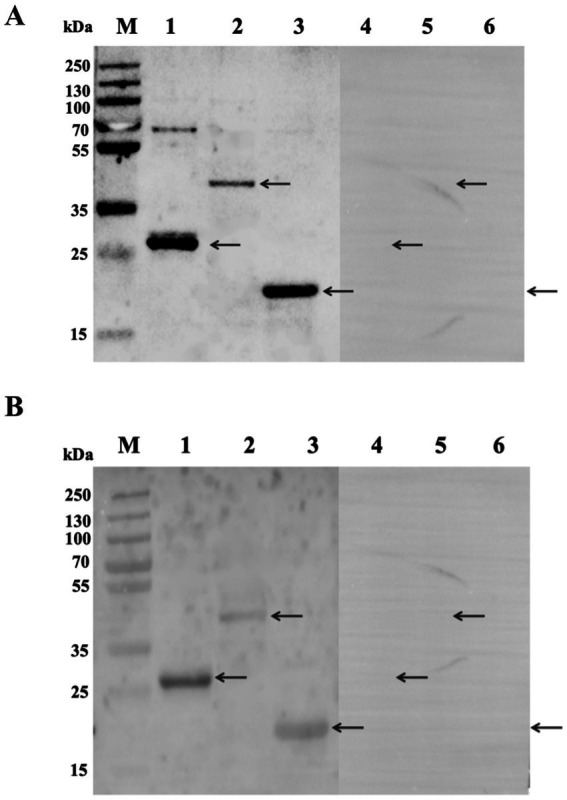
Analysis of the reaction of recombinant proteins with standard sera by Western blot method. **(A)** Reactivity of recombinant proteins with bovine sera. **(B)** Reactivity of recombinant proteins with sheep sera. Lane 1: rErfK reacts with positive sera; Lane 2: rYxeI reacts with positive sera; Lane 3: rEntF reacts with positive sera; Lane 4: rErfK reacts with negative sera; Lane 5: rYxeI reacts with negative sera; and Lane 6: rEntF reacts with negative sera. The arrows are a reference to the target protein bands.

The ability of these three Tat substrate proteins in diagnosing bovine and sheep brucellosis was evaluated using bovine and sheep positive and negative sera by the Western blot method. The results showed that the three Tat substrate proteins of *B. melitensis* M28 were recognized by the positive sera against *B. melitensis* and *B. abortus.* All tested proteins were not reacted with negative sera (Figure 3). Taken together, our results suggest that the Tat substrate proteins ErfK, YxeI, and EntF of *B. melitensis* M28 could be used in preparation of Tat substrates by ELISA.

### 3.3 Optimization of the tat substrate proteins’ concentration and serum dilution of iELISA

The optimal conditions for antigen coating and serum dilution in iELISA were determined by chessboard titration. Individual Tat substrate proteins and their combination were tested. The combination tests showed the best abilities in discerning bovine and sheep brucellosis-positive sera from negative sera, with maximum P/N ratios of 2.29 and 2.59, respectively. For the EntF group, the maximum ratio of P/N was 2.03 and 2.50, respectively, with an optimal dilution of serum being 1:400. ErfK and YxeI showed similar abilities to discern sheep brucellosis-positive and negative sera, with maximum P/N ratios of 2.37 and 2.33, respectively (Table 2).

**Table 2:** Optimization of antigen concentration and serum dilution for iELISA.

Antigen	Maximum P/N value	OD_450_ (mean ± SD) (positive serum)	OD_450_ (mean ± SD) (negative serum)	Antigen coating concentration (μg/mL)	Serum dilution
ErfK^a^	1.94	0.6862 ± 0.2877	0.3528 ± 0.1763	0.78	1:200
YxeI^a^	2.24	0.7363 ± 0.3046	0.3287 ± 0.1565	0.78	1:200
EntF^a^	2.03	0.5336 ± 0.1919	0.2625 ± 0.1299	0.78	1:400
Combination^a^	2.29	0.7808 ± 0.2699	0.3411 ± 0.11261	0.78	1:200
LPS^a^	1.49	0.7831 ± 0.1261	0.5256 ± 0.1038	1.00	1:200
ErfK^b^	2.37	0.5397 ± 0.5507	0.2274 ± 0.1190	0.78	1:200
YxeI^b^	2.33	0.5455 ± 0.5574	0.2345 ± 0.1138	0.78	1:200
EntF^b^	2.50	0.4900 ± 0.3905	0.1960 ± 0.0977	0.78	1:400
Combination^b^	2.59	0.5185 ± 0.4194	0.1999 ± 0.0977	0.78	1:400
LPS^b^	1.80	0.7046 ± 0.2621	0.3911 ± 0.1191	1.00	1:200

a Bovine sera.

b Sheep sera.

### 3.4 Comparison of tat substrate proteins in the serological detection of bovine and sheep brucellosis

Using brucellosis-positive and brucellosis-negative sera under the aforementioned optimal conditions, the efficiencies of individual Tat substrate proteins and their combination in serologically detecting bovine and sheep brucellosis were evaluated and compared.

**Figure 4:**
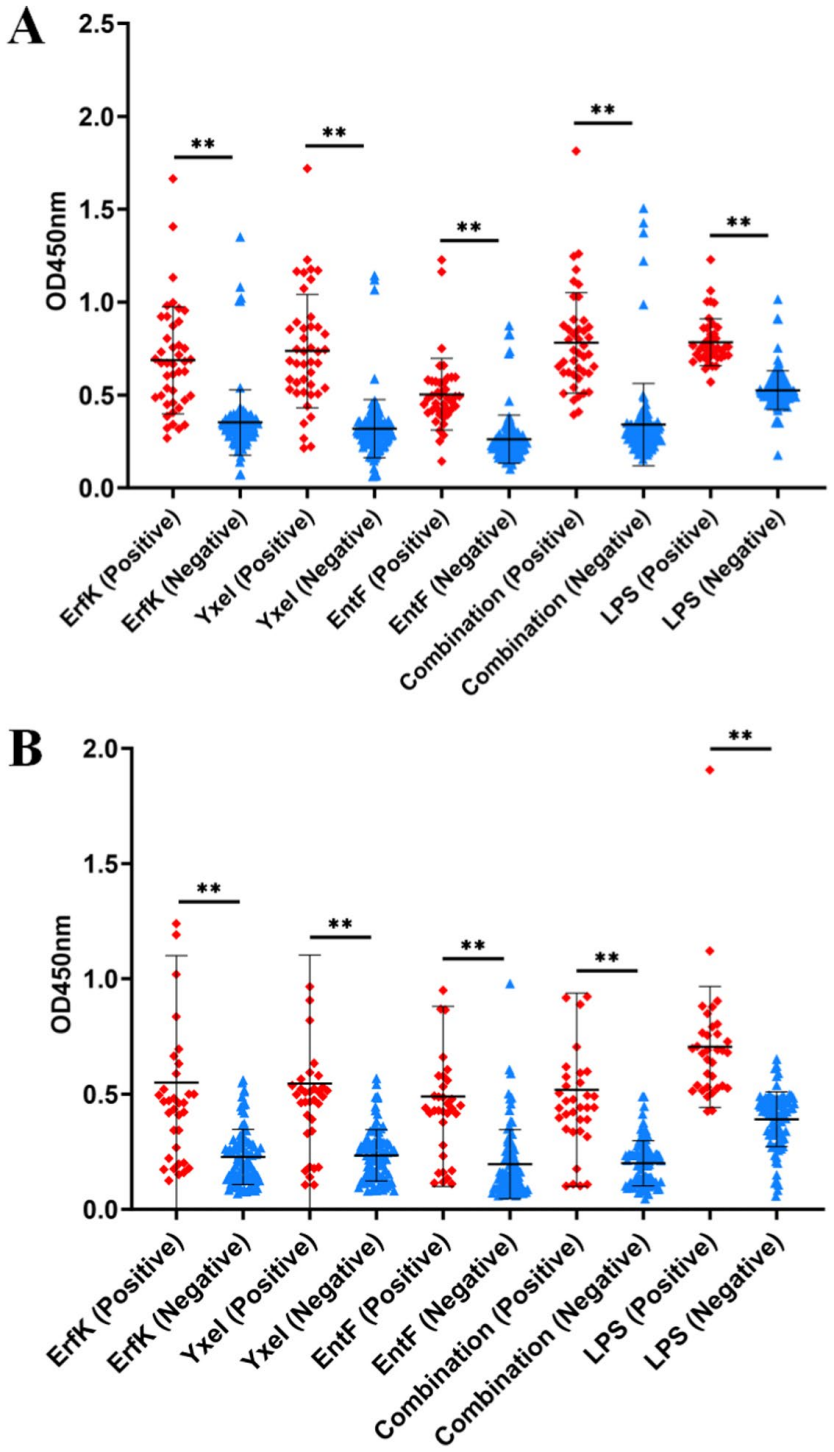
Indirect ELISA analysis of bovine and sheep samples. Dot plots of **(A)** bovine and **(B)** sheep samples.

**Figure 5:**
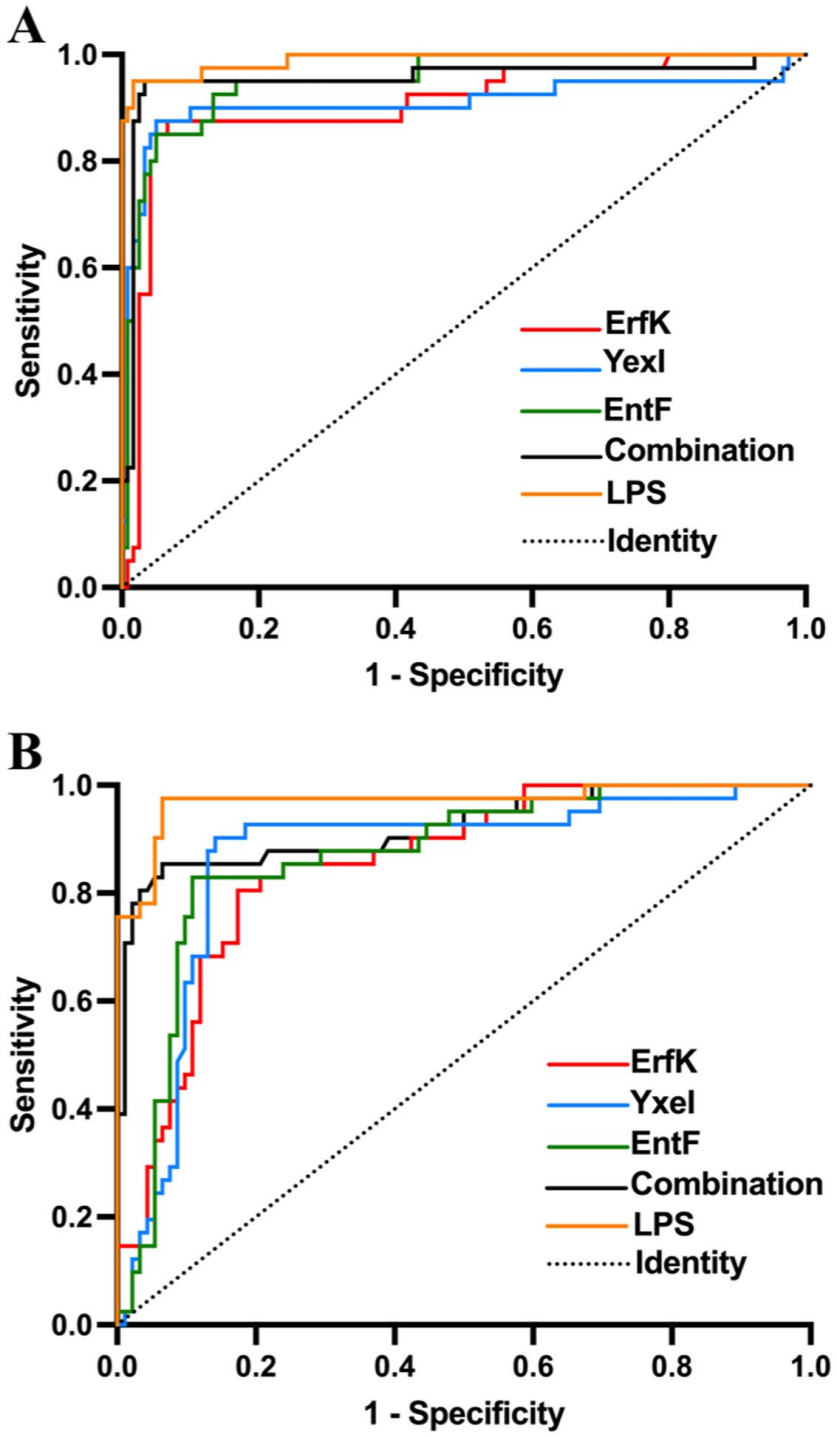
ROC analysis of rErfK, rYxeI, and rEntF in detecting bovine and sheep brucellosis. ROC analysis of **(A)** bovine brucellosis and **(B)** sheep brucellosis.

For the detection of bovine brucellosis, according to the iELISA results, the average OD_450_ values of 43 positive sera were 0.781, 0.686, 0.736, 0.503, and 0.783 for the combination and ErfK, YxeI, EntF, and lipopolysaccharides (LPS), while those of 113 negative sera were 0.341, 0.352, 0.318, 0.262, and 0.525, respectively (Table 3). The dot plot demonstrated that the combination and individual Tat substrate proteins ErfK, YxeI, and EntF showed difference in distinguishing bovine brucellosis-positive and negative sera (Figure 4A). The ROC curves were also obtained for these four antigens (Figure 5A). For the detection of bovine brucellosis, the AUC values of the combination, ErfK, YxeI, EntF, and LPS were 0.9549 (95% CI, 0.9190 to 0.9908), 0.8927 (95% CI, 0.8301 to 0.9553), 0.9121 (95% CI, 0.8474 to 0.9768), 0.9164 (95% CI, 0.8591 to 0.9738), and 0.965 (95% CI, 0.9354 to 0.9946), respectively. The combination showed improved accuracy and was superior to individual Tat substrate proteins alone. Based on the Youden index, the optimal cutoff value for the combination was 0.462. At the optimal cutoff value, 41 out of 43 positive samples were accurately diagnosed, and 106 out of 113 negative samples were accurately diagnosed. The positive predictive value (PPV) was 85.42%, and the negative predictive value (NPV) was 98.15%, making the combination the best performing antigen in serological diagnosis of bovine brucellosis. Correspondingly, the combination showed the highest accuracy (94.23%) in the serological detection of bovine brucellosis, similar to LPS (94.87%). The accuracies of individual Tat substrate proteins ErfK, YxeI, and EntF were 91.02, 93.58, and 91.02%, respectively (Table 3).

**Table 3:** Predictive values for positive and negative sera calculated at different cutoff values.

Cutoff value	Positive		Negative		PPV (%)	NPV (%)	Accuracy (%)
	TP	FN	TN	FP			
≥0.4057 (ErfK)^a^	37	6	105	8	82.22	94.59	91.02
≥0.4861 (YxeI)^a^	37	6	109	4	90.24	94.78	93.58
≥0.3426 (EntF)^a^	39	4	103	10	79.59	96.26	91.02
≥0.4620 (Combination)^a^	41	2	106	7	85.42	98.15	94.23
≥0.6368 (LPS)^a^	42	1	106	7	85.71	99.07	94.87
≥0.3447 (ErfK)^b^	24	10	79	13	64.68	88.76	81.75
≥0.3867 (YxeI)^b^	25	9	84	8	75.76	90.32	86.51
≥0.3694 (EntF)^b^	25	9	82	10	71.43	90.11	84.92
≥0.3131 (Combination)^b^	29	5	82	10	74.36	94.25	88.10
≥0.5053 (LPS)^b^	31	3	86	6	83.78	96.63	92.86

TP, true positive; TN, true negative; FP, false positive; FN, false negative; PPV, positive value (TP/TP + FP) × 100; NPV, negative predictive value (TN/TN + FN) × 100; and Accuracy, (TP + TN)/(TP + FN + TN + FP) × 100.

a Bovine sera.

b Sheep sera.

For the detection of sheep brucellosis, the average OD_450_ values of 34 positive sera were 0.518, 0.549, 0.545, 0.490, and 0.704 for the combination, ErfK, YxeI, EntF, and LPS, while those of 92 negative sera were 0.199, 0.227, 0.234, 0.195, and 0.391, respectively. The dot plot demonstrated that the combination and individual Tat substrate proteins ErfK, YxeI, and EntF showed diverse efficiency in distinguishing brucellosis-positive and brucellosis-negative sera (Figure 4B). The ROC curve analysis showed that the AUC values of the combination, ErfK, YxeI, EntF, and LPS were 0.8864 (95% CI, 0.7742 to 0.9585), 0.8008 (95% CI, 0.7088 to 0.8928), 0.8197 (95% CI, 0.7188 to 0.9205), 0.8378 (95% CI, 0.7588 to 0.9168), and 0.9019 (95% CI, 0.9081 to 0.9916), respectively. Based on the Youden index, the optimal cutoff value for the combination was 0.3131. The positive predictive value (PPV) was 74.36%, and the negative predictive value (NPV) was 94.25%, indicating the combination as the best performing antigen in serological diagnosis of sheep brucellosis. The accuracies of the combination, ErfK, YxeI, EntF, and LPS were 88.10, 81.75, 86.51, 84.92, and 92.86%, respectively (Table 3).

### 3.5 Cross-reactivity and repeatability with the tat substrate proteins

To verify whether Tat substrate proteins employed as diagnostic antigens possess cross-reactivity with other bacteria, we selected rabbit sera that were infected with other bacteria or immunized with zoonotic pathogens for a cross-reactivity test. The results showed that ErfK had cross-reactivity with *Salmonella* according to an S/N (OD_450_, sample/negative)>2.1, and the combination, YxeI and EntF, showed no cross-reactivity to the rabbit sera immunized with *Yersinia enterocolitica* O9, *Escherichia coli* O157:H7, *Mycobacterium tuberculosis*, *Vibrio cholerae*, *Legionella*, and *Salmonella*, exhibiting relatively good specificity (Table 4). Regarding the intra- and inter-assay precision, the coefficients of variation (CVs) of repeated tests within batches and between batches were confirmed to be less than 10% (Table 5), suggesting the good stability of Tat-iELISA.

**Table 4:** Specific cross-reactivity test results of the indirect ELISA.

Pathogens	ErfK		YxeI		EntF		Combination		LPS	
	OD_450_	S/N	OD_450_	S/N	OD_450_	S/N	OD_450_	S/N	OD_450_	S/N
*Yersinia enterocolitica O9*	0.1040	1.28	0.0768	0.78	0.0692	0.92	0.0585	0.67	0.3212	3.44
*Escherichia coli O157:H7*	0.1076	1.33	0.0727	0.74	0.0702	0.93	0.0857	0.99	0.3004	3.21
*Mycobacterium tuberculosis*	0.1350	1.66	0.0826	0.84	0.0689	0.91	0.0423	0.49	0.1321	1.41
*Vibrio cholerae*	0.0789	0.97	0.0721	0.74	0.0656	0.87	0.1083	1.24	0.1956	2.09
*Legionella*	0.1044	1.29	0.1031	1.05	0.0787	1.04	0.0832	0.96	0.1845	1.97
*Salmonella*	0.2068	2.55	0.2038	2.08	0.1391	1.85	0.1639	1.88	0.1087	1.16
*Negative*	0.0812	-	0.0980	-	0.0754	-	0.0870	-	0.0935	-

**Table 5:** Results of indirect ELISA reproducibility test.

Antigen	Intra-assay CV (%)		Inter-assay CV (%)	
	Positive serum	Negative serum	Positive serum	Negative serum
ErfK^a^	4.44	5.07	3.59	6.26
YxeI^a^	6.53	7.36	2.87	4.96
EntF^a^	6.04	8.65	3.33	7.85
Combination^a^	5.52	8.50	4.24	6.77
LPS^a^	6.41	7.48	5.29	6.92
ErfK^b^	6.27	6.13	4.80	8.51
YxeI^b^	5.99	6.28	3.78	6.59
EntF^b^	4.79	8.37	5.27	7.44
Combination^b^	6.50	7.89	5.86	8.03
LPS^b^	4.56	5.41	5.74	9.21

CV: coefficient of variation.

a Bovine sera.

b Sheep sera.

## 4 Discussion

Brucellosis is a globally widespread zoonotic disease transmitted from domestic animals to humans, posing a significant threat to animal husbandry and public health (23). In China, the primary *Brucella* species responsible for human infections are *B. melitensis*, *B. abortus*, *B. suis*, and *B. canis*, with *B. melitensis* and *B. abortus* accounting for 80–90% of total brucellosis cases; infections from other species are relatively uncommon (15, 16). It is essential to elucidate the occurrence and epidemic patterns of bovine and sheep brucellosis, as well as to accurately select detection methods to identify infected animals. While pathogen isolation and nucleic acid testing are important, serodiagnosis is prioritized in majority of the endemic areas due to its ease of implementation in limited clinical settings with minimal resources (17). The majority of the available serological tests target anti-LPS antibodies, which exhibit extensive cross-reactivity with other Gram-negative organisms, leading to ambiguous diagnoses of animal brucellosis (18). Research indicates that *Brucella* antigen proteins can effectively elicit both humoral and cellular immune responses, and serological detection methods based on these proteins demonstrate good specificity and sensitivity (19–21). Consequently, there is an urgent need to develop new serological methods that utilize antigen proteins other than those currently in use for detecting brucellosis in cattle and sheep.

In our previous study, we identified that the Tat substrate proteins ErfK (A0577), YxeI (A1479), and EntF (B0249) of *B. melitensis* M28 significantly contribute to *Brucella* virulence; however, the diagnostic potential of these proteins in bovines and sheep has not been systematically explored. In this study, we successfully expressed recombinant ErfK, YxeI, and EntF using a prokaryotic expression system and verified their diagnostic values in serum samples obtained from infected bovines and sheep. The Western blot analysis revealed that all three proteins were recognized by serum from bovines and sheep positive for brucellosis, confirming their potential as antibody detection targets. The results obtained from indirect enzyme-linked immunosorbent assay (iELISA) indicated that the diagnostic performance of these antigenic proteins varied across different host sera. Notably, the diagnostic value was higher for bovine serum samples than those from sheep. The combined use of these proteins yielded stronger diagnostic effects than the individual proteins, achieving a positive predictive value (PPV) of 41/48 (85.42%) and a negative predictive value (NPV) of 106/108 (98.15%), resulting in an overall accuracy rate of 94.23% for the detection of bovine brucellosis. Among the individual proteins, YxeI demonstrated superior accuracy over ErfK and EntF. Furthermore, the combination of the three selected Tat substrate proteins exhibited no cross-reactions with *Yersinia enterocolitica* O9 and *Escherichia coli* O157:H7. In future studies, the combined protein group and YxeI may serve as candidates for diagnostic antigens or subunit vaccine development.

Overall, this study is the first to evaluate and analyze the effects of Tat substrate proteins in the clinical diagnosis of brucellosis, thereby providing a reference for future research in the areas of brucellosis diagnosis and vaccine development. To our knowledge, at least 23 proteins are known Tat substrates in *B. melitensis*, although this study evaluated only 3 of them. Future research will continue to assess the role of other Tat substrate proteins in diagnosis of brucellosis.

## Data Availability

The original contributions presented in the study are included in the article/supplementary material, further inquiries can be directed to the corresponding authors.
